# Manufacturing an artificial arterial tree using 3D printing

**DOI:** 10.1016/j.heliyon.2024.e31764

**Published:** 2024-05-23

**Authors:** Wisam S. Hacham, Ashraf W. Khir

**Affiliations:** a Mechatronics Engineering Department, Al-Khwarizmi College of Engineering, University of Baghdad, Baghdad, Iraq; b Department of Engineering, Durham University, Durham, United Kingdom

**Keywords:** Artificial arterial tree, Catalyst solidification, In-vitro model, 3D printing, Silicone rubber, Mock ciculatory loop

## Abstract

Models of the arterial network are useful in studying mechanical cardiac assist devices as well as complex pathological states that are difficult to investigate in-vivo otherwise. Earlier work of artificial arterial tree (AAT) have been constructed to include some of the major arteries and their branches for in-vitro experiments which focused on the aorta, using dipping or painting techniques, which resulted in inaccuracies and inconsistent wall thickness. Therefore, the aim of this work is to use 3D printing for manufacturing AAT based on physiologically correct dimensions of the largest 45 segments of the human arterial tree. A volume ratio mix of silicone rubber (98 %) and a catalyst (2 %) was used to create the walls of the AAT. To validate, the AAT was connected at its inlet to a piston pump that mimicked the heart and capillary tubes at the outlets that mimicked arterial resistances. The capillary tubes were connected to a reservoir that collected the water which was the fluid used in testing the closed-loop hydraulic system. Young's modulus of the AAT walls was determined using tensile testing of different segments of various wall thickness. The developed AAT produced pressure, diameter and flow rate waveforms that are similar to those observed in-vivo. The technique described here is low cost, may be used for producing arterial trees to facilitate testing mechanical cardiac assist devices and studying hemodynamic investigations.

## 1 Background

Determining the precise physiological response of the human arterial tree or interaction with implanted devices may be difficult due to patient variability; for this reason in-vitro contolled investigations are often used. Further, studying the hemodynamuics performance of medical devices prior to clinical trials has a pre-requisite of in-vitro testing, and hence the need for healthy and/or diseased performing Artificial Arterial Tree (AAT). Therefore, manufacturing AAT closely resembling the physiological arterial tree is clearly desireable, especially if it is efficiently placed within a well-organized Mock Circulatory Loop (MCL).

The MCL is a significant in-vitro set up that mimics the arterial system, whose main benefit is that it does not require ethical approvals; in the first instance by-passes the need for in-vivo experiments. Furthermore, the cost for carrying out experimental work for the production and using MCL is likely to be less than in-vivo comparable procedures. This is particularly relevant in studying hemodynamic characteristics such as arterial distensibility, peripheral resistance, and pathological conditions such as arterial stenosis and aneurysm.

The pressure and flow waveforms obtained using MCL can be utilized to gain important cardiovascular hemodynamical variables, such as pulse pressure, flow rate and, wave speed. Additionally, several applications have been studied using MCL, such as the timing of intra-aortic balloon pump inflation [1], the pulmonary and systemic circulation fluid balancing [2], hemodynamic investigations of artificial heart valves [3], rotary blood pump unloading effect [4], biventricular assist devices [5], extracorporeal life support [6].

There are three different groups of MCL: 1) the mechanical model, 2) the numerical model, and 3) merging the mechanical and numerical models (the hybrid model) [7]. To simulate the in-vitro ventricles and cardiovascular system, mechanical MCLs often use hydraulic pumps to resemble the native heart, flexible tubes to resemble arteries and capillary tubes to simulate the resistance to flow. However, the fixed mechanical design limits the range of applications. On the other hand, the numerical MCL provides some flexibility to vary the complexity of the cardiovascular system using mathematical expressions, although its capacity maybe limited in describing physiological inticacies due to the lack of physical representations, particularly when devices are being tested. The hybrid group uses a real-time interface between hydraulic and numerical models, using robust and sophisticated computer algorithms, to aid studing the mechanical aspects of the cardiovascular system, which maybe too difficult to represent with either the mechanical or numerical models alone. Consequently, the hybrid mechanical MCL model became a popular test-bed for cardiac assist device. Cappon and colleagues [8] recently conducted a comprehensive review of the computational and experimental models of three types of MCLs used for testing cardiac assist devices, providing an understanding of the MCL's technological features and advances.

Shi et al. [9,10] introduced the building-up procedure and general structure of existing MCLs. They focussed on mechanical MCLs models. In their inclusive work, they presented a concise overview of the cardiovascular mechanics. Further, they offered the associated computational methodologies. Furthermore, they synopsized the literature search methodology. In the essential body of their review, they narrated the development of the three groups of MCLs. The reviews of Shi and Cappon presented current loops limitation, and proposed useful directions of future MCLs research.

Kolyva et al. [1] designed a mock circulatory system to replicate the physiological environment for studying the intra-aortic balloon pump; a widely used cardiac assist device. Their system comprised of an artificial left ventricle, connected to a 14 branch polyurethane compound aortic model and implemented the physiological distribution of compliance and terminal resistance with capillary tubes of different sizes. They connected the ends of the aortic branches to a common convey mimicking the venous system and an overhead reservoir providing atrial pressure. Their MCL produced physiological phasic aortic pressure and flow, showing the main features of counterpulsation. They mentioned that their system is verified to be convenient for intra-aortic balloon pump testing and with more enhancements it can be applied to investigate other hemodynamic problems and examine the dynamics of ventricular assist devices.

Swillens et al. [11] produced a model from a patient abdominal aortic aneurysm computer tomography scans, and manufactured with rapid prototyping with lost-wax casting strategies. Their silicon cast was manufactured and built into a silicon block of their model, incorporated and implanted into a 1:1 scale hydraulic bench model of human arterial system and left heart that developed in Ref. [12]. Their design comprised 37 arterial segments built up of a two-component silicon rubber that included tapered segments, manufactured in-house using painting techniques [13]; although admirable, controlling wall thickness remained a challenge.

In our earlier work [14] we investigated the effect of aneurysms and stenoses on wave reflection in single conduits, using similar methods to those presented in the current study. The previous encouraging results laid the groundwork for producing a more extensive and sophisticated MCL. Given the need for MCLs and recent global interest in replacing animal experiments with in vitro testing, it is advantageous to produce MCL that closely mimics the in-vivo arterial bed. Therefore, the aim of the present work is design and manufacture an AAT of 45 segments based on dimensions of a healthy human [15], which as far as the authors are aware, has not previously been manufactured. We also aim to present the novel methodology used in building the MCL.

## 2 Tools, materials, and methodologies

Manufacturing the AAT involve sequential steps, which are outlined as follows:

*Design and planning*: starting with conceptual design including the dimensions, branching patterns, and the overall complexity required for this specific application. Preparaton of the computer-aided design (CAD) software for creating the detailed blueprint.

*Material selection*: choosing a suitable material for constructing the AAT. Considering factors such as flexibility, durability, transparency (if required), and ease of manipulation.

*3D printing or mold fabrication*: depending on the complexity of the arterial tree model, it can be employed 3D printing techniques to create the structure directly or use a mold-based approach. 3D printing can offer precise control over the design, while molds allow for replication.

*Parts assembly*: developing a suitable assembly process to connect the individual arterial segments. This can involve hand or specialised machinery capable of precise positioning and bonding of the segments.

*Integration of fluidic channels*: incorporating channels within the arterial tree model to simulate blood flow. These channels can be designed to mimic the dimensions and flow characteristics of real arteries. Depending on the material used, it can either embed the channels during 3D printing or create them separately and integrate them into the model.

*Quality control*: implementing quality control measures to ensure the accuracy and functionality of the manufactured arterial tree model. This can involve visual inspection, pressure testing, flow simulation, and other relevant assessments.

*Iterative refinement*: refining the manufacturing process based on feedback and improvements identified during initial testing. Iterating the design and manufacturing steps to enhance the model's performance and capabilities.

*Documentation and validation*: document the manufacturing process and validation results to establish a comprehensive record. This documentation can be useful for replication, sharing with the scientific community, or regulatory compliance if the model is intended for medical applications.

The following sub-sections describe in details the various elements used in constructing the MCL, including the quantification of Young's modulus of the AAT walls using tensile testing.

Step [1]: 3D printing of the molds.

**Fig. 1:**
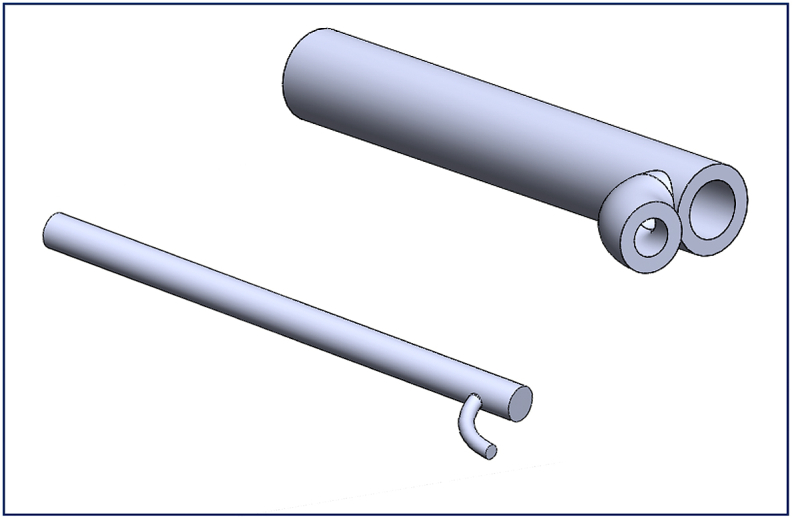
Illustrates the configuration of the male (lower) and female molds (upper) of the right ulnar artery and its bifurcation. SolidWorks software was used to draw this schematic. All the dimensions are shown in Table 1.

**Fig. 2:**
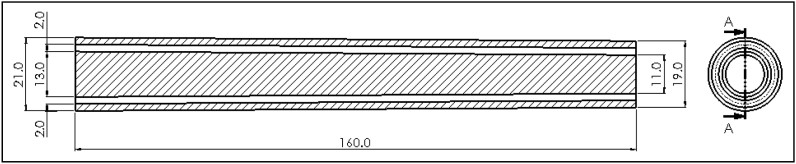
Technical drawing for the assembled 3D printed mold of the abdominal aorta. All dimensions shown are in (mm) as shown in Table 1.

SolidWorks software (SolidWorks Corp., Dassault Systèmes, 2019, Massachusetts Institute of Technology, USA) was used to produce the technical drawings of all n = 45 vessels of the AAT. As an example, Fig. 1 depicts the configuration of the male and female molds of the right ulnar artery and its bifurcation.The physiological dimensions of the AAT segments including the length, the proximal and distal cross-sectional area of each segment were taken from Ref. [15]. The cross-sectional area was assumed to vary linearly over the short length of the artificial segment, and data of all segments are included in Table (1). STereoLithography files (STL) were generated by three-dimensional design and uploaded to 3D printer (The solid imaging company, Viper si2 SLA system, UK, Hemel Hempstead) to produce a blend, durable, and flexible SLA 3D printed molds using (easycomposites, CS2 condensation cure silicone rubber, UK). The material used in fabricating the SLA 3-D molds (Accura SI 10 polymer) is a light-reactive thermoset material of epoxy-resin and hardener. Additive manufacturing to create objects through sequential layering, by exposing a liquid polymer to a digitalized ultra-violent laser projector, was the principle of a 3D printing machine performed in the present work. The final configuration of printed molds was gained under safelight conditions. Some of the molds have a smaller diameter compared to its length which made the injection process difficult, especially for the peripheral arteries. Therefore, those molds have been divided into more than one segment to facilitate the printing. An example of the 3-D printed sub-segments molds following assembly in Fig. 2 that shows the technical drawing of the assembled abdominal aorta. SolidWorks was used to facilitate the 3D design as follows.

*Conceptualization and sketching*: we began by conceptualizing the two-dimensional design of the arterial arterial tree and its bifurcations. Then we used SolidWorks to sketch out the basic shapes and structures of the male and female parts of molds.

*3D modeling*: we used SolidWorks' 3D modeling tools to convert our sketches into three-dimensional models. Hence we created individual parts of the mold, such as the main arteries, arterial bifurcations, aortic arch, and other necessary parts.

*Assembly*: we assembled the individual male and female parts to produce the final artery's mold assembly using SolidWorks' assembly features. Then we ensured precise fitting and alignment between all parts of the artery's assembly.

*Draft analysis*: we analyzed the draft angles on the mold using SolidWorks to ensure easy ejection of the molded parts. This adjustment was performed using a 3D-printed base. This printed base guaranteed precise alignment for all parts within a mold, preventing any spillage of the mixture from the side opposite to where it is poured. When the mold stands vertically with one side open and the other sealed by this base, which stays in contact with the platform, it ensures containment and proper positioning during the pouring process.

*Detailing and documentation*: we wrote the necessary details, such as part numbers, annotations, and dimensions, on the mold's outer surface. These detailed drawings and documentation were critical during the manufacturing process to ensure accurate connection between adjacent parts without errors that might alter the internal diameter of these arteries, and crucial to avoid unrealistic arterial expansion or constriction. Such discrepancies could lead to inaccurate outcomes that impact result quality.

*File export and collaboration*: we exported designs in STL format using SolidWorks to be compatible with the 3D printer used.

*Iterative process and refinement*: we used SolidWorks' flexibility to make iterative changes based on the gained design. We refined the inner surface of the female parts and the outer surface of the male part for each mold to smooth them for better performance and manufacturability and to prevent mechanical problems, such as cracks and tears, during the step of grabbing the printed part from the mold.

Although SolidWorks offers simulation and analysis tools to validate the design for functionality, strength, and mold flow analysis to ensure mold design that is capable of handling the molding process effectively without defects, we did not do the step of design validation and testing. Because any mold consists of two parts (male and female), and the female part is formed by cutting two identical parts lengthwise, the molds would not endure significant stresses from pouring the rubber mixture during or after casting. Additionally, dividing the long arteries into two parts aims to ease the process. Manual printing guarantees 3D-printed arteries without potential mechanical issues such as voids in the dried mold or bubble formation, which could compromise the mechanical efficiency of the artery and lead to failure during testing. This approach also minimizes the likelihood of exposing the molds to high mechanical stresses causing their defects. Finally, before pouring the mixture into the molds, the alignment of the two segments of the female part was achieved during the printing process. This was done by utilizing regular adhesive tape after filling the space between them with Bluetack along the length.

Step [2]: AAT production.

Liquid silicone was added to catalyst [easycomposites CS2] with 2 % volume ratio to facilitate solidifying the mixture and form silicon rubber which was manually injected into the gap between the male and female of the molds. When cured, the liquid mixture formed the arterial walls with thickness of 2 mm. Manual injection of the silicon rubber in the gap between the male and female of each segment needed to be carried out speedly to avoid the mixture drying up mid-process.

**Table 1:** Geometrical data for the artificial arterial model [15].

No.	Segment	Length (mm)	Proximal diameter (mm)	Distal diameter (mm)
1	Ascending Aorta	40	30.0	29.0
2	Aortic Arch	60	29.0	21.0
3	Thoracic Artery	160	21.0	13.0
4	Abdominal Aorta	160	13.0	11.0
5	Right common iliac Artery	58	07.5	07.0
6	left common iliac Artery	58	07.5	07.0
7	Right external iliac Artery	145	06.0	05.5
8	left external iliac Artery	145	06.0	05.5
9	Right femoral Artery	450	05.5	04.0
10	Left femoral Artery	450	05.5	04.0
11	Right posterior tibial Artery	330	05.0	03.0
12	Left posterior tibial Artery	330	05.0	03.0
13	Right Anterior tibial Artery	330	05.0	03.0
14	Left Anterior tibial Artery	330	05.0	03.0
15	Right deep femoral Artery	125	05.2	04.0
16	Left deep femoral Artery	125	05.2	04.0
17	Right internal iliac Artery	50	04.0	04.0
18	Left internal iliac Artery	50	04.0	04.0
19	Right subclavian Artery	250	08.5	08.0
20	Left subclavian Artery	180	08.5	08.0
21	Right brachial Artery	280	08.0	05.0
22	Left brachial Artery	280	08.0	05.0
23	Innominate Artery	34	13.0	13.0
24	Right vertebral Artery	150	03.76	02.66
25	Left vertebral Artery	150	03.76	02.66
26	Right common carotid Artery	210	07.40	07.4
27	Left common carotid Artery	210	07.40	07.4
28	Right internal carotid Artery	180	03.6	01.6
29	Left internal carotid Artery	180	03.6	01.6
30	Right external carotid Artery	180	03.6	01.6
31	Left external carotid Artery	180	03.6	01.6
32	Right radial Artery	235	03.5	02.8
33	Left radial Artery	235	03.5	02.8
34	Right ulnar Artery	240	04.3	03.6
35	Left ulnar Artery	240	04.3	03.6
36	Right interosseous Artery	80	01.8	01.8
37	Left interosseous Artery	80	01.8	01.8
38	Celiac Artery	20	07.8	04.0
39	Hepatic Artery	66	04.4	04.4
40	Gastric Artery	71	03.6	03.6
41	Splenic Artery	63	05.5	05.5
42	Right renal Artery	32	05.0	05.0
43	Left renal Artery	32	05.0	05.0
44	Superior mesenteric Artery	58	08.7	08.7
45	Inferior mesenteric Artery	50	03.2	03.2

Step [3]: connecting AAT segments.

Following curing, the individual segments had been connected by gluing using liquid silicone, cautiously ensuring no entry of silicon into the segemental lumen to keep inner surfaces of the walls smooth without abnormalities. The two ends of the segments have been carefully turned several times to ensure the silicon glue was applied sufficiently to the entire circumference. This step was particularly tedious in producing bifurcations for gluing the daughter tubes to the parent vessel.

Step [4]: connecting the capillary arteries.

The terminals of the arterial tree have been linked with artificial capillary tubes to obtain the terminal resistances required for the representation of the systemic circulation. To do so, Poiseuille's law was adopted with the no-slip condition and data published by Stergiopulos, 1992. Briefly, For the steady flow through tubes in a sufficient length with a constant cross-section area, the flow will be fully developed and, therefore, the pressure gradient will be constant. The terminal resistance (R) with respect to Poiseuille law, $R=8\mu L\;{\left(\pi r^{4}\right)}^{-1}$, where (μ) is dynamic viscosity, (L) is the length and (r) is the radius of the capillary tube.

**Fig. 3:**
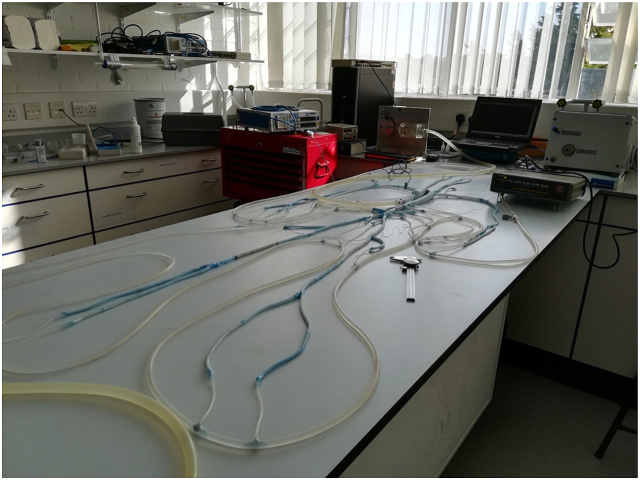
Shows the artificial arterial segments connected together, and the peripheral arteries connected to the artificial venous tube via capillary tubes, all collectively presenting the artificial arterial tree.

Based on the above equation and using values of the terminal resistances that were published in Steriopulos, 1992, the values of radius and length of capillary tubes used in the present work were calculated as shown in Table 2, following earlier work by Kolyva et al. [1]. The capillary tubes were connected to a plastic tube that mimics the venous return as shown in Fig. 3 which illustrates the two steps mentioned above.

**Table 2:** Terminal resistances data due to capillary tubes, [dynamic viscosity= (0.8903*10^−3^ Pa s) at 25^0^C of the laboratory temperature].

Segment	Resistance [N.sec.m^−5^]	Capillary tube radius [mm]	Capillary tube length [mm]
11,12,13 and 14	0.4770E+10	0.5	131.5
15 and 16	0.4770E+10	0.5	131.5
17 and 18	0.7936E+10	0.5	218.8
24 and 25	0.6010E+10	0.5	165.7
28,29,30 and 31	0.1390E+11	0.5	383.2
32 and 33	0.5280E+10	0.5	145.6
34 and 35	0.5280E+10	0.5	145.6
36 and 37	0.8430E+11	0.25	145.2
38	0.3630E+10	0.5	100.0
39	0.2320E+10	0.75	322.38
40	0.5410E+10	0.5	149.1
41	0.2320E+10	0.75	322.38
42	0.1130E+10	0.75	157.7
43	0.1130E+10	0.75	157.7
44	0.9300E+10	0.75	129.8
45	0.6880E+10	0.5	189.7

Step [5]: Pump and Measurements.

A useful utility of the AAT is to be used as a mock circulatory loop, hence a reciprocating piston pump, home manufactured with a bore of 5 cm and stroke length of 5 cm, was used to produce pulsatile flow mimicing the native heart and connected to the ascending aorta. A control unit of piezo-crystal probe [Sonometrics digital ultrasonic transducer, trx series 4, Canada, Ontario] was used to measure the diameter signal at the ascending aorta. Also, a flow meter (HT323; Transonic System Inc, Ithaca, NY, USA) with the appropriate probe size (32 mm) was used for measuring the flow rate at the ascending aorta. A pressure transducer-tipped catheter (Gaeltec, Scotland, UK) was used to measure the pressure at the ascending aorta. Further, the system used water as the flowing fluid and reservoirs were used for providing and collecting the water. Data were sampled at 500Hz using National Instruments (BNC-2090 DAQ, TX, USA).

### 2.1 Tensile testing

**Fig. 4:**
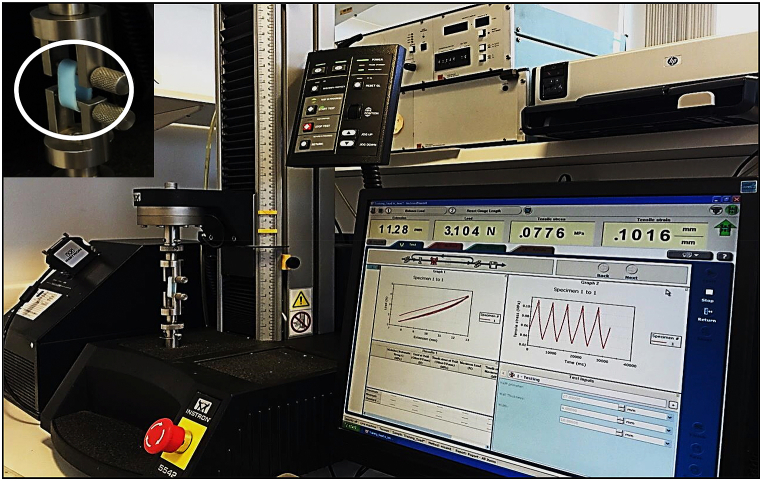
Setup of Instron machine that used to perform the tensile tests for the manufactured silicone parts.

Tensile tests were performed to estimate Young's modulus of elasticity for the walls of the manufactured silicone parts. A tensiometer (Instron High, Wycombe, UK) was used for testing hoops of the different tubes that were used for constructing the AAT. Fig. 4 shows the setup of the tensiometer with a specimen, and also shows graphically the force-extension data for determining Young's Modulus.

We performed an initial warm-up on the manufactured samples that underwent tensile testing. This involved stretching and relaxing the samples five times successively to confirm the absence of any tearing or defects originating from the printing process that might influence the resulting modulus of elasticity values. At least 3 experiments were carried out on the identical segments (100 mm width and 20 mm inner diamter).

## 3 Results

### 3.1 Pulsatile testing of AAT

**Fig. 5:**
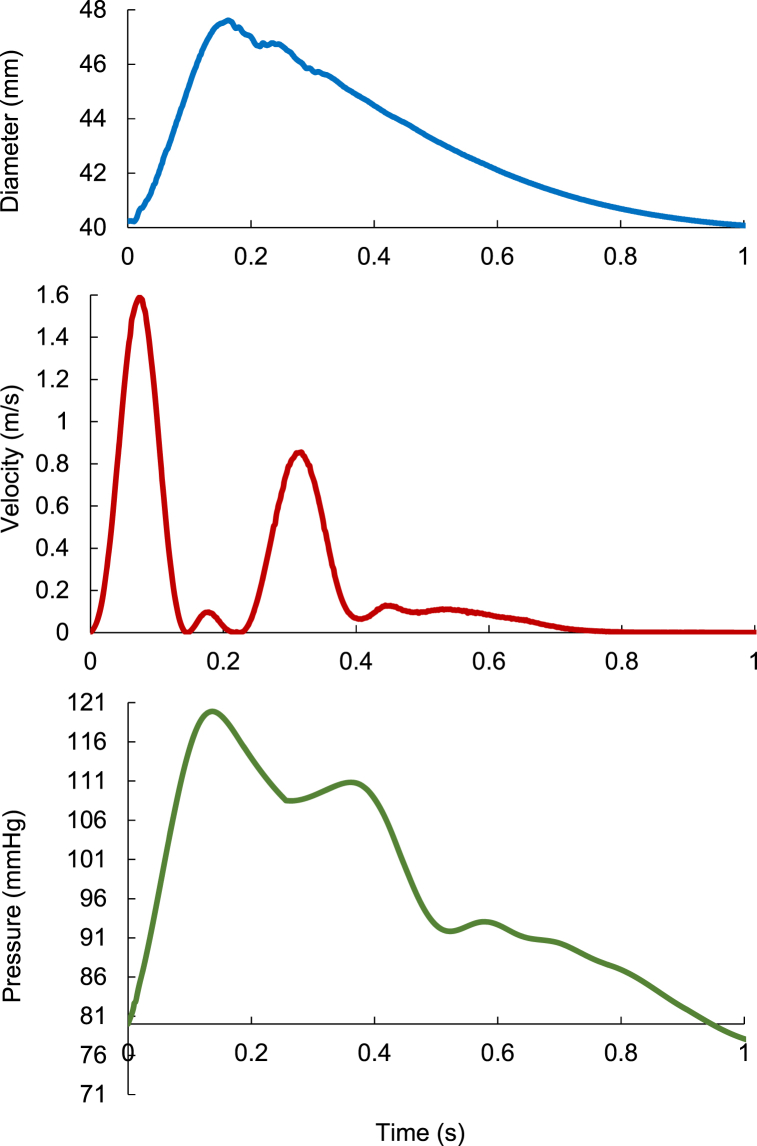
Measurements of diameter signal (upper), velocity wave (middle), and pressure wave (lower) at the ascending aorta for AAT. Sample time interval is 0.003. The pulse time was approximately equal to 1 s. The diameter signal collected using Sonometrics transducer [Control unit of piezo-crystal probe]. The velocity acquired using ultrasound flowmeter and probes (Transonic, HT323, NY, USA). The pressure wave measured using catheter transducer (Transducer-tipped catheters, Gaeltec, Scotland, UK). The 2nd order Savitzky-Golay filter [16] was employed to smooth waveforms.

One of the main goals of producing the AAT was to provide a testbed that mimics the physiological arterial tree. To demonstrate its functionality of producing physiological waveforms, we collected pressure, diameter and flowrate waveforms in the AAT. Fig. 5 shows smaple waveforms measured at the ascending aorta. The readings were taken five times for each of the recorded signals producd. The shape and size of the waveforoms are evidently in good agreement with those measured in-vivo. The inclusion of physiological waveform measurements aimed to showcase the tree's capability to replicate physiological wave patterns, thus validating our proposed manufacturing method.

Our AAT was manually self-manufactured, including all the main arteries and bifurcations. Fig. 2 shows the tapering fashion of the arterial vessel; the decrease in size of the aorta with distance from the ascending aorta.

### 3.2 Young's modulus determination

Young's modulus for the 1 mm and 2 mm wall thickness was 1.8 ± 0.15 MPa and 1.3 ± 0.12 MPa, respectively. Table 3 shows the results of the tensile testing for all specimens used in the present work. All results consistently aligned, affirming the reproducibility of the AAT, and its intended objectives.

**Table 3:** Report the results of tensile testing. All silicone rubber specimens are 10 mm of width and 20 mm of inner diameter. The testing performed at temperature of 20.3^**0**^C and humidity of 35 kg/m^**3**^.

Segment Thickness (mm)	Young's Modulus (MPa)	Load at Yield (N)	Tensile stress at Yield (MPa)	Maximum Load (N)
2.0	1.30 ± 0.12	63 ± 5.0	1.60 ± 0.15	64 ± 2.2
1.5	1.50 ± 0.12	38 ± 3.5	1.30 ± 0.13	39 ± 2.1
1.0	1.80 ± 0.15	28 ± 3.5	1.27 ± 0.10	33 ± 2.1
0.75	1.95 ± 0.15	25 ± 3.5	1.22 ± 0.11	28 ± 2.1
0.50	2.11 ± 0.18	21 ± 3.0	1.10 ± 0.11	18 ± 2.3

## 4 Discussion

This work presents the first mock circulatory loop of physiologically correct dimensions for the largest 45 segments of the arterial system, featuring the physiological tapering, using 3D printing techniques. Our previous paper focused on examining the impact of arterial aneurysms and stenosis on wave intensity, wave reflection, and speed within these discontinuities [14]. The methodology used to create these discontinuities are similar to those utilized in constructing the arterial tree presented in this current study. The extended intricate details of the current methodology and incorporating an AAT with 45 segemnts stand as the novelty of the current paper. Further, for the purpose of validating the MCL presented in the work, we used a a computer-controlled mechanical pump, to replicate the heart, and took measurements of pressure, flow velocity and diameter and produced waveforms that are comparable to physiological data.

Table 3 shows the results of the testing the specimen of 1 mm wall thickness. The elasticity for this specimen is 1.8 ± 0.15 MPa is greater than that of the specimens of 2 mm and 1.5 mm wall thickness. Similarly, Young's modulus of the wall of a vessel in 20 mm of diameter with 0.75 and 0.5 mm of thickness are 1.95 ± 0.15 MPa and 2.11 ± 0.18 MPa, respectively. Our tests indictae that a decrease in wall thickness leads to an increase in the elasticity of the tube wall.

The catalyst was added to facilitate the solidification of the liquid silicone to form the silicone rubber material. The time required to solidify the liquid silicone without the use of the catalyst could be as long as 30 days. To examine the effect of the added quantity of the solidification on the elasticity of manufactured silicone rubber specimens, two specimens with different catalyst/silicone ratios were manufactured and tested. A 2 % ratio resulted in elasticity of 1.3 MPa and a ratio of 5 % resulted in unphysiologically low elasticity. Consequently; it can be confirmed that increasing of the catalyst/silicon ratio leads to a decrease in the elasticity of the segment wall, and whilst using a catalyst can not be avoided, the mixture ratio should not exceed 2.5 % to produce segments with physiological values. However, creating pathologicall-like segments with low elsaticity such as those observed in marfan syndrome can be produced by varying wall thickness and the catalyst/silicone ratio. Using similar techniques, Hacham and Khir [14] produced artificial arterial segments with stenosis and aneurysms which presented discontinuity to wave travel resembling those observed in-vivo. Such segments can be easily added/integrated to the current AAT to study the effect of discontinuity on the hemodynamic signals and wave intensity patterns.

The ability of this MCL and AAT to accurately capture hemodynamic waveforms and demonstrate good agreement with physiological data is indeed a significant achievement. This suggests that the model may effectively assess abnormalities such as stenosis (narrowing of blood vessels) and aneurysms (ballooning of blood vessel walls) in the context of pulse wave propagation and mechanical cardiac assist devices.

Having an accurate and reliable AAT model is valuable for several reasons. It can serve as a tool for studying and understanding the complex hemodynamics of the cardiovascular system. By simulating blood flow and pressure patterns, insights could be gained into how different pathologies affect the arterial system. Further, the model can be used for testing and evaluating the performance of mechanical cardiac assist devices. These devices are designed to assist or replace the pumping function of the heart and can have a significant impact on blood flow dynamics. Using the AAT model provides means to assess how such devices interact with the arterial system and potentially optimize their design and functionality. Overall, the successful capture of hemodynamic waveforms and agreement with physiological data by the AAT model is a promising development in the field of cardiovascular research and medical device engineering. It provides confidence that the model can be a valuable tool for assessing abnormalities and studying the effects of various interventions on the arterial system.

The consistency of the AAT wall depends on the length of the hand-made segment. The longer the segment, the more difficult it is to print hence the more difficult it is to get a isotropic wall. The greater the length of the piece, the greater the possibility of air bubbles forming that would eventually form tears/cracks in the manufactured arterial wall, which would in turn affect the mechanical properties of the wall, and consequently cause poor measurements/outcome. To avoid air bubbles trapment within the formed pieces, the right mixture was lifted to a certain height to ensure that gravity forces overcame the forces of the friction between air and the mixture on the surface tension to push air bubbles upwards. The pieces could also be placed on a standard laboratory shaker to assist air bubbles escapy out of the mixture.

The liquid-based material used in fabricating the SLA 3-D molds is a light-reactive thermoset material of epoxy-resin and hardener (SLA resin, UK) suited the printer we utilized. Newer materials such nylon (Selective Laser Sintering SLS), Acrylonitrile Butadiene Styrene (ABS), polyester (Polylactic Acid PLA) are also available and could be used to provide similar quality of the models. A cost-benefit analysis could be conducted to ensure each application uses a reasonably priced material without compromising the objectives and quality required. A promising use of water-soluble 3D printer filament such as Polyvinyl Alcohol (PVA) (1.75 mm) would enable the inner mold to dissolve, eliminating the need for physical disassembly or having to compromise the quality of the final product.

Using 3D printing technology has currently become at the heart of a considerable research in the medical field due to its capabilities in mimicking human organs and tissues. The enormous potential of these techniques aids in precise modelling [17], constructing customised prostheses and implants that pertain to the anatomy or defects of bio-host organs and tissues [[18], [19], [20]], patient-specific anatomy models constructing [21,22]. This enhances the competence to produce controlled structures of artificial organ or tissue features that have efficient biological characteristics [23,24]. Furthermore, providing 3D-printed pharmaceutics biomimetic constructs for drug screening [[25], [26], [27]]. Recently, numerous applications of 3D-printed cardiac surgery, using qualitative cardiac models, have been reported [28,29]. Lately, 3D printing techniques have offered the capability to make significant assistance in mitigating medical products shortages, including the latest coronavirus pandemic [30].

The 3D silicone model developed by Swillens et al. [11] was constructed using qualitative imaging of computer tomography scans, which is a comparatively expensive technique requiring data from multiple volunteers to ensure accuracy. They employed semi-automatic segmentation and the mimics software package (Materialise, Leuven, Belgium) to validate their model's surface reconstruction, necessitating significant expertise in software. Manufacturing their model involved costly rapid prototyping and lost-wax casting techniques, requiring professional skills. They created a silicone cast incorporated into a silicone block (RTV 282, Polyester Demaere, Ledeberg, Belgium) to ensure sufficient stiffness and muscularity capable of withstanding physiological pressures. Achieving the desired in-vitro model elasticity within the reported range of in-vivo elasticity required multiple tests for their thick-walled model. Their artificial arterial model comprised 37 arterial segments, including tapered segments like the aorta, which were manufactured in-house using a painting technique with two-component silicon rubber (Baysilone LSR2050, General Electric, Fairfield, CT). However, the absence of the lower part of the arterial tree may impact the results, particularly in terms of realistic peripheral resistance and accurate venous pressure representation. They connected their manufactured segments using silicone material, except for the rigid connection between their AAA silicone assembly and the remaining model segments, potentially affecting wave reflection from the aneurysm discontinuity. In contrast, our methodology allows for the merging of a discontinuity into the arterial tree without requiring rigid ligation. Comparatively, their methodology is more expensive and involves extensive arrangements and handling compared to the approach we followed. The comparison of speed waves collected from the two teams reveals close similarities, considering that the comparison was made with their work before utilizing AAA-repair.

3D printing has brought positive changes to various fields, including biomedical engineering. The ability to replicate vital parts of the human body using 3D printers has indeed revolutionized the way researchers obtain/regenrate data and predictions without the need for surgical intervention. Additionally, the progress in biological materials and biomaterials has further enhanced these capabilities. With 3D printers and their capabilities, researchers and medical professionals have been able to create prosthetic limbs, customized implants, and even intricate models for surgical planning, further assiting in the potential management of patients. Moreover, the growth of 3D printing and its impact on material and cognitive expansion aligns well with the global objective of improving quality of life. As this technology continues to develop, it has the potential to revolutionize manufacturing, construction, and many other industries, leading to more sustainable and efficient urban environments.

With the rapid pace of scientific progress and the ongoing advancements in this field, it's reasonable to expect even more impressive results in the future. Indeed, any advancement in 3D printing technology will contribute to cognitive urbanization growth.

## 5 Limitation

This work used manufactured silicone parts of the produced AAT resulted in different compliance than that of arteries in-vivo. Arriving at the exact in-vivo compliance will require a different mix ratio but is not expected to change the captured waveforms significantly. The methodology presented in this work is not time-efficient, and further work is required to automate the process.

The discrepancy in compliance between the manufactured silicone parts used in this study and the actual in-vivo arterial compliance poses a limitation. Achieving precise compliance would require altering the mix ratio between the silicon and the catalyst, a difficult balancing act as it has a direct effect on the curing duration. Regardless, while this adjustment may refine accuracy, it is not anticipated to have significant effect on the waveforms. Further, the current approach demands substantial time investment, signaling a need for further exploration toward an automated process. The manual nature of the methodology hinders its scalability and applicability or mass production of the MCL.

The AAT manufactured in this study is based on dimensions of an average individual, as reopreted in the literature [15], and therefore may not fully encapsulate the complete spectrum of biological variability present in human arterial systems. Variations in arterial dimensions, wall thickness, and material properties among different individuals or specific arterial segments might not be accurately replicated using this methodology. Thus, the model's dimensions will need to change depending on the intended human cohort representation and on the physiological conditions observed in-vivo.

The model's representation of the arterial bed using silicon walls might not entirely capture the dynamic interplay of physiological factors observed in living arterial systems. Biological arteries exhibit dynamic responses to various stimuli, including changes in blood pressure, flow patterns due to biochemical influences. The current techniques for manufacturing arterial tree might not fully account for these dynamic interactions, potentially limiting the accurate reproduction of physiological responses in real-time. Similarly, While efforts have been made to validate the manufactured arterial tree through waveform measurements, replicating the entire range of physiological conditions and scenarios observed in-vivo remains outstanding. Factors such as temperature variations, pulsatile flow dynamics, and posture (angle to the horizontal) need to be taken into consideration using an auto regulation models; a future direction of our work.

## 6 Conclusions

The technique proposed in this work for fabricating a 3D-printed artificial arterial tree, is cost effective but not time efficient. The use of the artificial arterial tree is beneficial in studying the characteristics of cardiovascular system, testing of mechanical ventricular assist devices and allows for in-vitro credible approch to studying the left side of the arterial system.

Using the current silicon/catalyst volume ratio confirms that elasticity is inversely proportional to the wall thickness of the flexible tubes and the smaller the thickness of the AAT, the larger its modulus of elasticity.

The flexibility of using the procedure and methodology described in this work for producing the AAT is a function of the silicone rubber, which in turn depends on the silicone/catalyst volume ratio. The smaller the volume ratio (greater catalyst), the smaller the elasticity of the AAT wall. The larger the volume ratio (greater silicone), the longer the cure time of the mixture.

Finally, replicating the baroreflex responses of the in-vivo arterial bed is one of our targets for future studies. These explorations can pave the way for advancing this field and potentially reducing the necessity for animal experiments for studying cardiac and arterial dysfunctions.

## Data availability

Data will be made available on request.

## CRediT authorship contribution statement

**Wisam S. Hacham:** Writing – original draft, Visualization, Validation, Software, Resources, Methodology, Investigation, Formal analysis, Data curation, Conceptualization. **Ashraf W. Khir:** Writing – review & editing, Validation, Supervision, Resources, Methodology, Conceptualization.

## Declaration of competing interest

The authors declare that they have no known competing financial interests or personal relationships that could have appeared to influence the work reported in this paper.
